# Technique originale de relèvement enclouage à foyer fermé d'une fracture thalamique du calcanéum

**DOI:** 10.11604/pamj.2015.22.179.6816

**Published:** 2015-10-22

**Authors:** Soufiane Guelzim, Noureddine Sekkach

**Affiliations:** 1 Service de Chirurgie Orthopédique et Traumatologie, CHU Ibn Sina, Rabat, Maroc; 2 Service de Chirurgie Orthopédique et Traumatologie, Hôpital Delafontaine, Saint Denis, France

**Keywords:** Calcanéum, enclouage, fluoroscopie, Calcaneum, nailing, fluoroscopy

## Image en medicine

Le relèvement enclouage à foyer fermé (R.E.F.F) est une technique originale de traitement des fractures thalamiques du calcanéum. Il s'agit d'un patient âgé de 40 ans sans antécédents, victime d'une chute domestique sur le talon droit, d'une hauteur de 3 mètres. Le patient présentait un ‘dème et une ecchymose du talon droit, sans ouverture cutanée. L'examen vasculo-nerveux était normal. Le bilan radiologique a objectivé une fracture thalamique du calcanéum droit type IV (Duparc) avec un angle de Böhler négatif; fracture à 4 fragments avec un enfoncement mixte horizontal et vertical. Nous avons réalisé la technique de relèvement enclouage à foyer fermé (R.E.F.F) sous contrôle fluoroscopique: patient installé en décubitus latéral gauche, sous rachianesthésie. Réduction et ostéosynthèse percutanée à l'aide de 2 clous de Steinmann (A, B): 1 clou relevant la surface thalamique et restituant l'angle de Böhler et le 2^ème^ clou corrigeant le varus de la grosse tubérosité (C, D). Traitement chirurgical complété par une immobilisation plâtrée pendant 6 semaines. Les Suites opératoires ont été simples; ablation du plâtre et des clous de Steinmann à 6 semaines, et une rééducation a été entreprise. L'appui partiel a été autorisé à 8 semaines, appui total à 3 mois. Le résultat fonctionnel était bon avec un recul de 12 mois.

**Figure 1:**
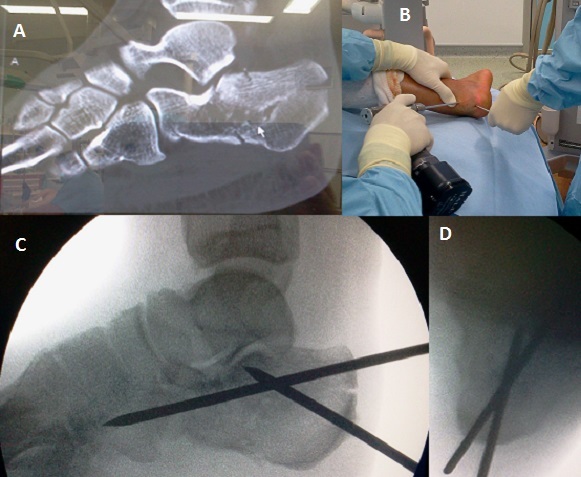
(A) scanner: fracture thalamique mixte à 4 fragments; (B) image per opératoire; (C) contrôle fluoroscopique peropératoire; (D) contrôle fluoroscopique peropératoire: incidence rétro-tibiale

